# Restriction on antimicrobial dispensing without prescription on a national level: Impact on the overall antimicrobial utilization in the community pharmacies in Saudi Arabia

**DOI:** 10.1371/journal.pone.0271188

**Published:** 2022-07-26

**Authors:** Ahmed Hamdan Al-Jedai, Yasser Almogbel, Khalid Eljaaly, Nasser M. Alqahtani, Hajer Yousef Almudaiheem, Nancy Awad, Dema Abdulrahman Alissa, Abdullah Assiri, Tareef Alaama

**Affiliations:** 1 Therapeutic Affairs Deputyship, Ministry of Health, Riyadh, Saudi Arabia; 2 Colleges of Pharmacy and Medicine, Alfaisal University, Riyadh, Saudi Arabia; 3 Department of Pharmacy Practice, College of Pharmacy, Qassim University, Buraydah, Saudi Arabia; 4 Clinical Pharmacy, Faculty of Pharmacy, King Abdulaziz University, Jeddah, Saudi Arabia; 5 Riyadh First Health Cluster, Ministry of Health, Riyadh, Saudi Arabia; 6 Clinical Pharmacy, College of Pharmacy, Alfaisal University, Riyadh, Saudi Arabia; 7 Real World Evidence, IQVIA, Dubai, United Arab Emirates; 8 Assistant Deputy Ministry of Public Health, Ministry of Health, Riyadh, Saudi Arabia; Public Library of Science, UNITED KINGDOM

## Abstract

**Background:**

High rates of non-prescription dispensing of antimicrobials have led to a significant increase in the antimicrobial overuse and misuse in Saudi Arabia (SA). The objective of this study was to evaluate the antimicrobial utilization following the enforcement of a new prescription-only antimicrobial dispensing policy in the community pharmacy setting in SA.

**Methods:**

Data were extracted from the IQVIA database between May 2017 and May 2019. The antimicrobial utilization rates, based on sales, defined daily dose in grams (DDD), DDD/1000 inhabitants/day (DID), and antimicrobial-claims for the pre-policy (May 2017 to April 2018) and post-policy (June 2018 to May 2019) periods were assessed.

**Results:**

Overall antimicrobial utilization declined slightly (~9–10%) in the post-policy versus pre-policy period (sales, 31,334 versus 34,492 thousand units; DDD, 183,134 versus 202,936), with higher claims (~16%) after policy implementation. There was a sudden drop in the utilization rate immediately after policy enforcement; however, the values increased subsequently, closely matching the pre-policy values. Utilization patterns were similar in both periods; penicillin was the most used antimicrobial (sales: 11,648–14,700–thousand units; DDD: 71,038–91,227; DID: 2.88–3.78). For both periods, the highest dip in utilization was observed in July (sales: 1,027–1,559 thousand units; DDD: 6,194–9,399), while the highest spike was in March/October (sales: 3,346–3,884 thousand units; DDD: 22,329–19,453).

**Conclusion:**

Non-prescription antimicrobial utilization reduced minimally following policy implementation in the community pharmacies across SA. Effective implementation of prescription-only regulations is necessary.

## Introduction

Recent advancements in newer and more potent antimicrobials have immensely changed the infectious disease treatment landscape, playing a critical role in the overall improvement of public health. However, their inappropriate utilization over the years has led to the emergence of multi-drug resistant (MDR) pathogens, which have the potential to pose a serious threat to humanity [1–4]. The incidence of antimicrobial-resistant infections is on a continuous rise around the globe, contributing to poor healthcare outcomes, increased mortality rates, and high healthcare costs [5–7]. About 2.8 million people are estimated to be infected with MDR infections each year causing more than 35,000 deaths in the US [8], and 256,000 associated deaths in North Africa and Middle East [9]. Per published reports, a patient dies every 10 minutes due to these deadly superbug infections in the developed countries such as the US or Europe [10]. A report by the World Health Organization (WHO) highlights the growing antibiotic resistance rates throughout the Eastern Mediterranean Region [11].

In Saudi Arabia (SA), the overall prevalence of antimicrobial resistance (AMR) among Gram-negative and Gram-positive organisms was >25% during 2007–2016 and >15% during 2015–2019 [12, 13], and has one of the highest rates of these difficult-to-treat infections [2], probably related to the high utilization of antimicrobials in the Middle East countries [1, 2, 14, 15]. Irrational prescribing and dispensing practices are extremely high in SA, in addition to the increased rate of self-medication with over-the-counter (OTC) antimicrobials (approximately 50–80%) [1, 2]. Likewise, high sales of antimicrobials without prescriptions have been reported in the United Arab Emirates (UAE; ~68%) [16]. Surveys of the community pharmacies in Riyadh, Jeddah and Madinah highlight the extremely high use of non-prescription antimicrobials (~98%) and the increasing tendency among community pharmacists to sell antimicrobials without a prescription [17–19]. A report published in 2017, further highlights the wide-spread use of non-prescribed antimicrobials, unveiling the extensive unethical dispensing practices in pharmacies across SA [16]. These prescribing attitudes of pharmacists in retail pharmacies, emphasize the lack of awareness of the implications of overuse of these life-saving drugs and disregard for regulatory guidelines and requirements [18].

Relentless efforts have been made by the SA health authorities to curb the looming catastrophe of antimicrobial misuse/overuse in the country. In this regard, the Bureau of Experts at the Council of Ministries has enforced stringent laws and regulations that explicitly prohibit the pharmacists from dispensing antimicrobials without a prescription from a physician [16]. However, despite robust regulations and legislations for the accessibility to antimicrobials, their unethical use remains a glaring concern in SA. Therefore, as a strategy to address this unsettling issue, the Ministry of Health (MoH) enacted the enforcement of the Executive Regulations of Health Practice Law in May 2018 that restricts dispensation of antimicrobials only on a legal prescription [20]. Pharmacists who violate the law are liable to be punished with a fine of up to 27,000 USD, imprisonment for up to 6 months, and revoking of their pharmacy license. This conforms to the tactical approach adopted by many countries across the globe, such as, Mexico, Brazil, Colombia, Venezuela and Chile [21–23], to develop their own policies encompassing legislative changes regarding prescription requirements, reimbursement restrictions and professional regulations.

The objective of this quasi-experimental study was to evaluate the actual antimicrobial utilization by estimating their sales and defined daily dose (DDD) per 1000 inhabitants per day (DID) [24] in the community pharmacy setting across SA, following the implementation of the new policy framework. Our study was based on the hypothesis that the utilization of antimicrobials measured in the number of sales, DDD and number of claims would decline after implementation of the new policy.

## Materials and methods

### Study design and data collection

In this study, retrospective data were extracted from IMS Health (now IQVIA) between May 2017 and May 2019. IMS Health is a for-profit agency that gathers information on drug sales, health services and technologies. We collected data on total sales of antimicrobials in the private retail sector (pharmacies across SA) to assess their actual utilization nationwide, during the period from one-year before (pre-policy: May 2017 to April 2018) to one year after (post-policy: June 2018 to May 2019) enforcement of the policy (May 2018). The month of enforcement was excluded from the pre- and post-policy estimation. The claims data were collected following the same period from three main private payers in SA, contributing to almost 80% of the private sector.

### Inclusion criteria

Antimicrobial utilization data during the study period mentioned above only was included. The study data was restricted to the dispensation of antimicrobials by prescription only. Analyses were restricted to oral antimicrobials in all dosage forms (tablets, suspensions, and capsules). Sales, DDD and claims data were collected for the following antimicrobials (predominantly antimicrobials): amoxicillin, amoxicillin-clavulanic acid, azithromycin, clarithromycin, ciprofloxacin, levofloxacin, moxifloxacin, clindamycin, cefuroxime, cefdinir, cephalexin, sulfamethoxazole/trimethoprim, doxycycline, minocycline, metronidazole, fluconazole, oseltamivir and nitrofurantoin.

### Exclusion criteria

Parenteral (intravenous/intramuscular), topical, ophthalmic, otic, and local (intravaginal and pessaries) antimicrobial data were excluded from the study. Data gathered outside the study period were not included.

### Metrics for antimicrobial utilization

The primary outcomes were to evaluate the difference in the utilization of antimicrobials, as measured by the sales volume in the pre- and post-policy periods, to determine the DDD, and to assess the overall impact of policy on the claims. The DDD data were expressed as DDD per 1000 inhabitants per day (DID), and for DID estimation the population/inhabitant estimate was used as the denominator [24]. The overall antimicrobial sales volume is expressed as number of antimicrobial units sold. The secondary outcomes included, studying the correlation between the sales volume and DDD between similar months in the pre- and post-policy periods.

Overall antimicrobial utilization based on DDD was calculated as follows:

$$Antimicrobial\;DDD=\frac{No.\;of\;packages\;\times \;No.\;of\;tablets\;per\;package\;\times \;No.\;g\;per\;tablet}{WHO\;DDD\;of\;antimicrobial\;in\;grams}$$

### Statistical analysis

As all the sales and claims within the defined pre- and post-policy periods were included, and as sample size normality was assumed based on the Central Limit Theorem (CLT), the sample size was not calculated in this study. Descriptive analyses were used to identify the frequency of sales, DDD and claims of different antimicrobials. A Regression Discontinuity Design (RDD) was used to assess the effect of the new policy (dispensing the antimicrobials only with prescriptions) on sales and utilization. The sales data were coded according to the Anatomical Therapeutic and Chemical (ATC) classification [25]. A subgroup analysis was performed to assess the antimicrobial sales and utilization by chemical class and molecule. Data analysis was performed using SAS (Statistical Analysis Software) and R softwares.

### Ethics statement

The study protocol was approved by the institutional review board, MoH Research Ethics Committee [26]. This study did not involve the collection, use, or transmittal of individual identifiable data, yet the study involved the use of anonymized patient level data represented by the claims as well IQVIA sales data, hence the need for consent was waived by the ethics committee.

## Results

**Fig 1:**
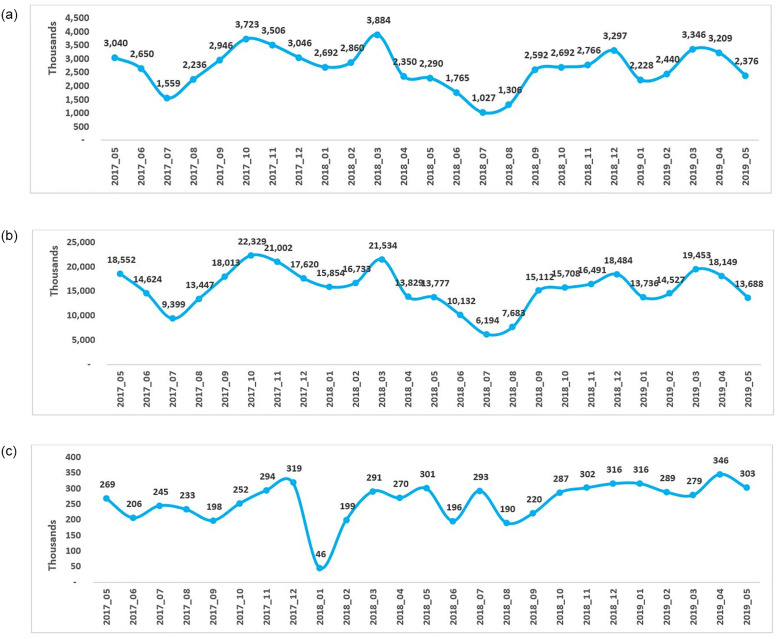
Overall sales and utilization and number of claims of antimicrobials in the year pre-and post-policy enforcement in Saudi Arabia. A] Antimicrobial sales volume (units), B] Antimicrobial utilization (DDD) and C] Number of antimicrobial claims. For each month in the pre-policy period (May 2017-April 2018), post-policy period (June 2018 to May 2019) and policy enforcement (May 2018), the overall antimicrobial sales volume which is the number of antimicrobial units sold (A), antimicrobial utilization (DDD) (B), and number of antimicrobial claims (C) were presented. DID, defined daily dose.

The overall antimicrobial sales volume (number of antimicrobial units sold), utilization (DDD) and claims decreased following implementation of the policy in May 2018 (Fig 1A–1C). The absolute sales volume and utilization for the pre-policy period (May 2017 to April 2018) were 34,492 thousand units and 202,936, respectively, while for the post-policy period (June 2018 to May 2019), the numbers decreased to 31,334 thousand units and 183,134, respectively. An overall decrease of 3,154 thousand units (~9%) in the sales volume and a reduction in the utilization by 19,803 (~10%) was observed post-policy compared to the pre-policy period.

Consistently, DID values decreased by ~17% in the post-policy period (6.27 DID) compared to the pre-policy period (7.59 DID), indicating a reduction in the use of antimicrobials after policy enforcement (see Table 1). Comparison of the data of similar months for the pre- and post-policy periods and the corresponding percentage difference indicated a decrease in the overall sales volume by 12–42%, and DDD by 13–42% in the post-policy period compared to pre-policy period (except for the months of December 2018 and April 2019 when a spike in sales and DDD was noted) (Fig 1A and 1B).

**Table 1:** Antimicrobial utilization (DID) by chemical class and molecule in the year pre- and post-policy enforcement in Saudi Arabia.

A] DID by chemical class		
	Pre-policy period (May 2017 to April 2018)	Post-policy period (June 2018 to May 2019)
Penicillin (J01C)	3.78	2.88
Trichomonacides (G01A)	1.66	1.54
Fluoroquinolones (J01MA)	0.65	0.56
Macrolides and lincosamides (J01F)	0.43	0.36
Cephalosporins, carbapenem and monobactam (J01D)	0.35	0.29
Tetracyclines (J01A)	0.28	0.25
Anti-infective agents (urinary) (G04A)	0.16	0.08
Anaerobicides (J08B)	0.11	0.08
Systemic antifungal agents (J02A)	0.08	0.09
Trimethoprim combinations (J01E)	0.07	0.05
Antiviral agents (J05B)	0.01	0.08
Amebicides (P01A)	0.01	0.01
**Total**	**7.59**	**6.27**
**B] DID by molecule**		
Amoxicillin, Clavulanic Acid	4.54	3.93
Amoxicillin	3.02	1.81
Metronidazole	1.77	1.61
Cefuroxime Axetil	1.70	1.40
Azithromycin	1.45	0.98
Ciprofloxacin	1.22	1.00
Clarithromycin	0.94	0.92
Doxycycline	0.80	0.67
Levofloxacin	0.44	0.39
Moxifloxacin	0.24	0.24
Fluconazole	0.08	0.09
Cefalexin	0.08	0.03
Sulfamethoxazole trimethoprim	0.07	0.04

DID, defined daily dose per 1000 inhabitants per day

The yearly trend of antimicrobial sales and utilization remained largely similar for both the pre-policy and post-policy periods. The line graphs for sales volume and DDD for both periods showed a trough between June and August, indicating a dip in sales and utilization of antimicrobials, with the lowest numbers observed in July (absolute values: pre-policy, July 2017 –sales = 1,559 thousand units, DDD = 9,399; post-policy, July 2018 –sales = 1,027 thousand units, DDD = 6,194) (Fig 1A and 1B). Further, there were two peaks observed in the sales volume and DDD graphs for both the periods, indicating high sales and utilization during these months, with the highest numbers reported in the month of October 2017 and March 2018 in the pre-policy period (sales = 3,723 and 3,884 thousand units and DDD = 22,329 and 21,534 in October 2017 and March 2018, respectively). Similarly, highest numbers were reported in December 2018 and March 2019 in the post-policy period (sales = 3,297 and 3,346 thousand units and DDD = 18,484 and 19,453 in December 2018 and March 2019, respectively) as shown in Fig 1A and 1B. Another observation that may be of relevance, was the most drastic drop after a peak in April 2018 (pre-policy period) with respect to the immediately preceding month, March 2018, in both sales (~43% decrease) and DDD (~38% decrease) (Fig 1A and 1B). Compared with similar months in the post-policy period, *viz*. April 2019, the decline observed was minimal from the previous month, March 2019 (~4% and ~6% decline in sales and utilization, respectively) (Fig 1A and 1B).

We used claims data for one major payer only, as the data for the other two payers were inadequate and did not support any relevant interpretation. The yearly trend for claims was consistent during both the periods, pre- and post-policy. The number of antimicrobial claims showed an overall increase (~16%) after policy implementation (Fig 1C). The total number of antimicrobial claims in the pre-policy period was 2,882 thousand, and in the post-policy period was 3,337 thousand. Notably, a drastic decrease in the number of claims was observed in June 2018 (absolute value: 196 thousand), immediately after the implementation of the policy in May 2018 (absolute value: 301 thousand). Surprisingly, there was a sudden decline (~86%) in claims in January 2018 (pre-policy period) from the previous month. However, no such effect was observed in the post-policy period in January 2019. Additionally, a slight dip in the number of claims was noted in April 2018 from the previous month (~7% decrease) in the pre-policy period. Comparatively, in the post-policy period, there was an increase observed in the number of claims in April 2019 (~24% increase from the previous month).

For each month in the pre-policy period (May 2017-April 2018), post-policy period (June 2018 to May 2019) and policy enforcement (May 2018), the overall antimicrobial sales volume which is the number of antimicrobial units sold (1A), antimicrobial utilization (DDD) (1B), and number of antimicrobial claims (1C) were presented. DID, defined daily dose.

**Fig 2:**
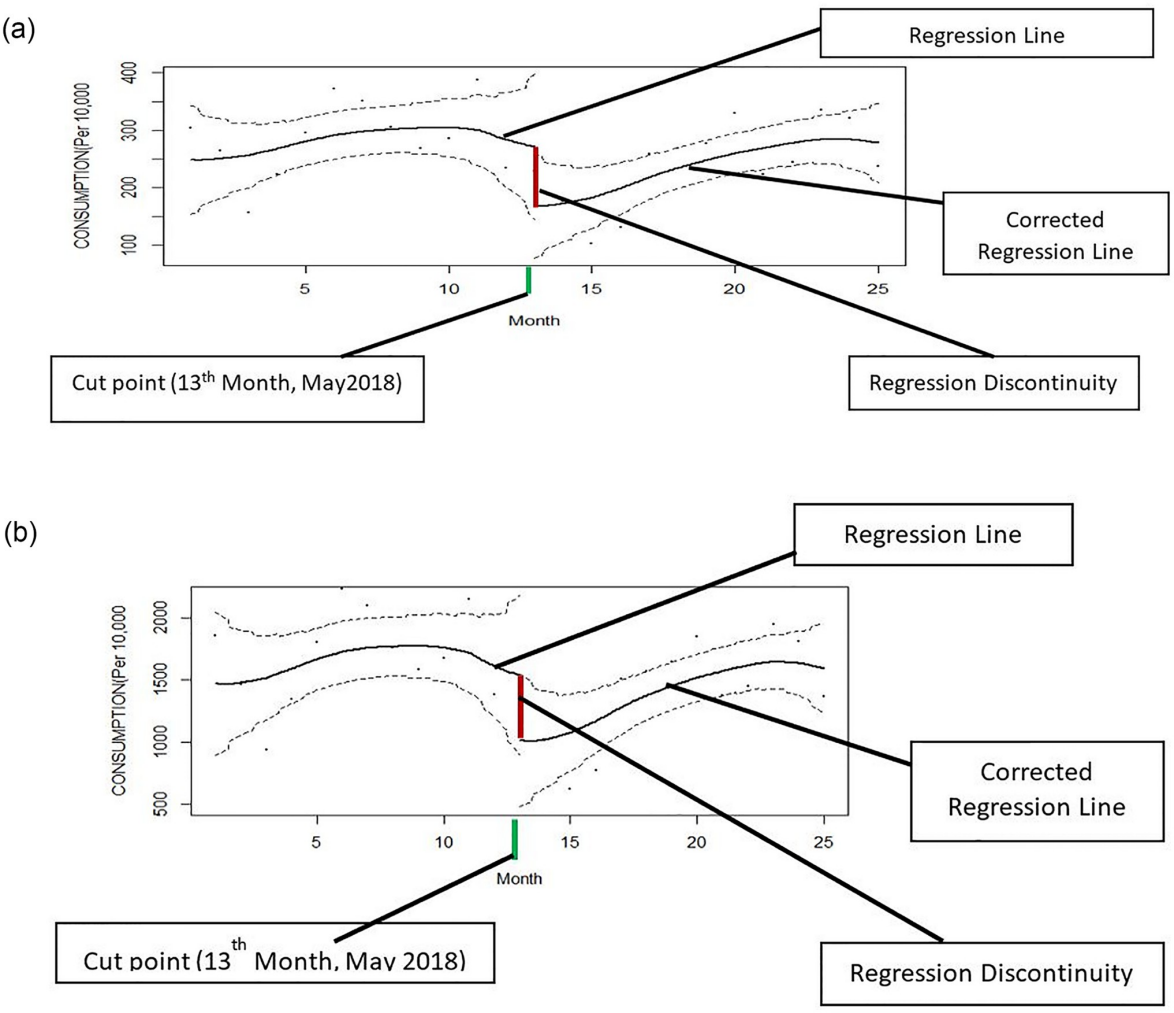
Effect of an intervention based on regression discontinuity design. A] Sales volume, B] Define daily dose (DDD).

The results of RDD analysis were also in line with the above findings, highlighting a sudden decline in the sales and utilization of antimicrobials, immediately after the implementation of the policy (Fig 2A and 2B). After the sudden decline post implementation of the policy, both parameters started to increase later during the post-policy period.

**Fig 3:**
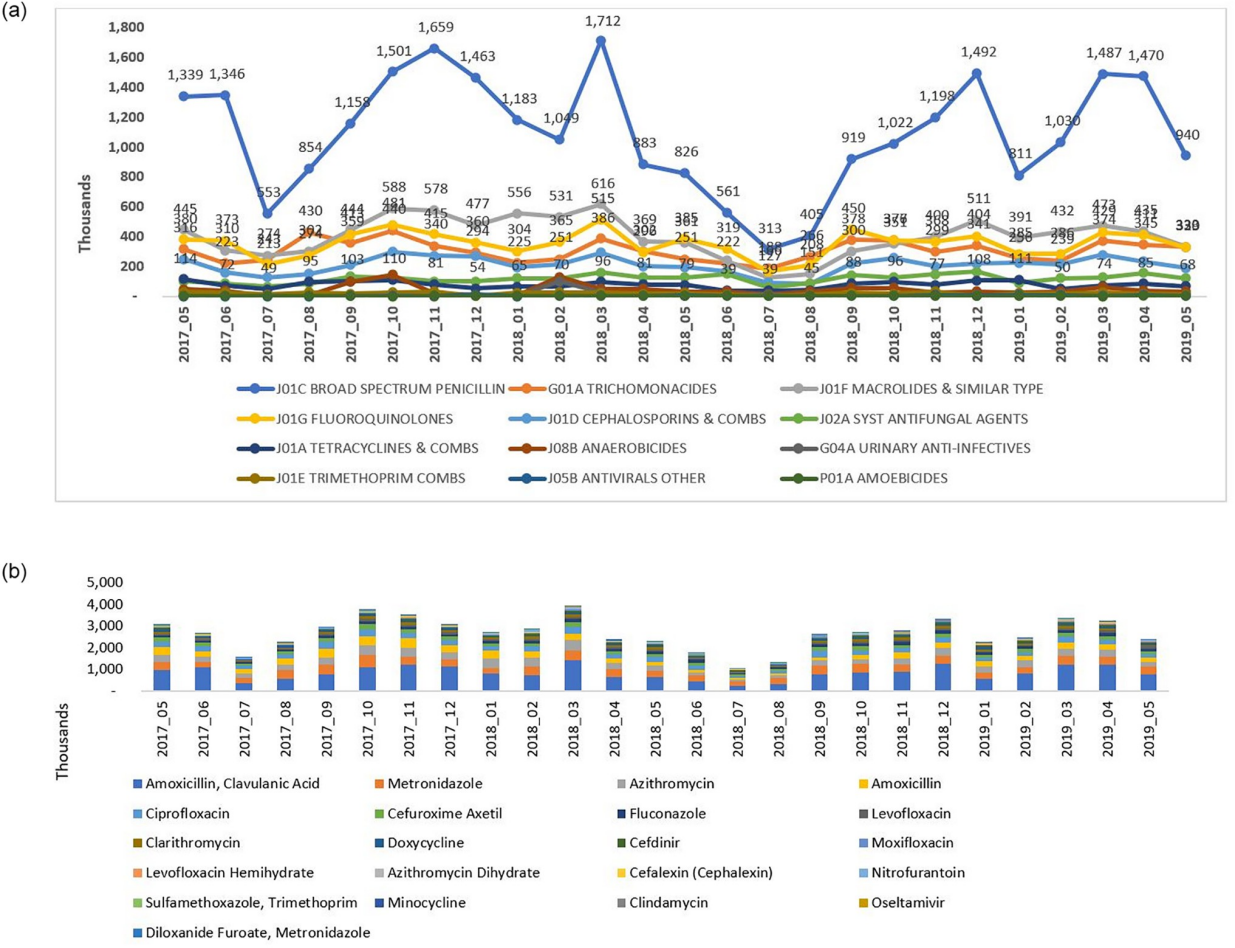
Antimicrobial sales volume in the years pre-and post-policy enforcement in Saudi Arabia. A] Antimicrobial sales by chemical class (ATC3 classification), B] Antimicrobial sale by molecule.

**Fig 4:**
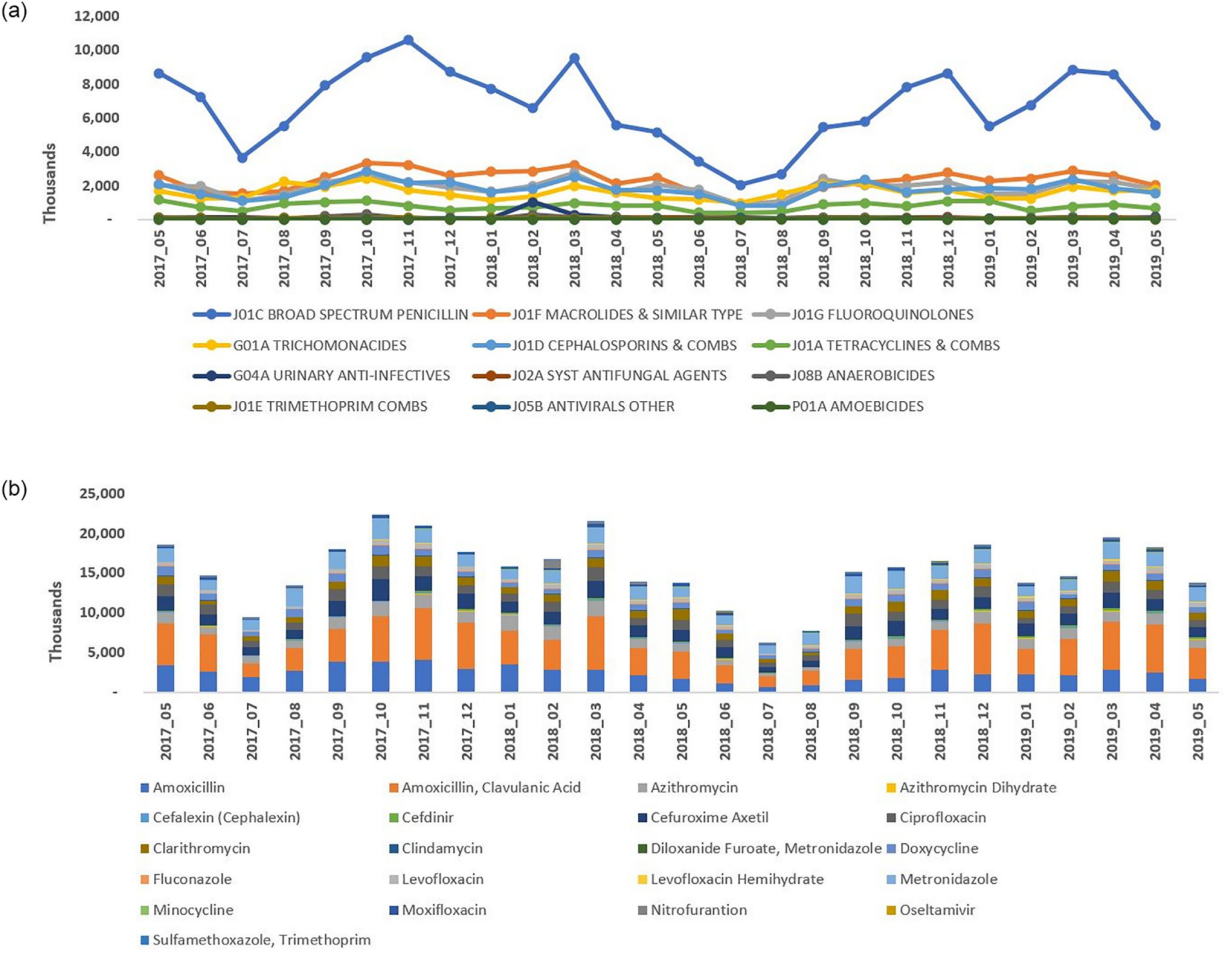
Antimicrobial utilization (DDD) in the year pre- and post-policy enforcement in Saudi Arabia. A] Antimicrobial utilization by chemical class, B] Antimicrobial utilization by molecule. DDD, defined daily dose.

**Fig 5:**
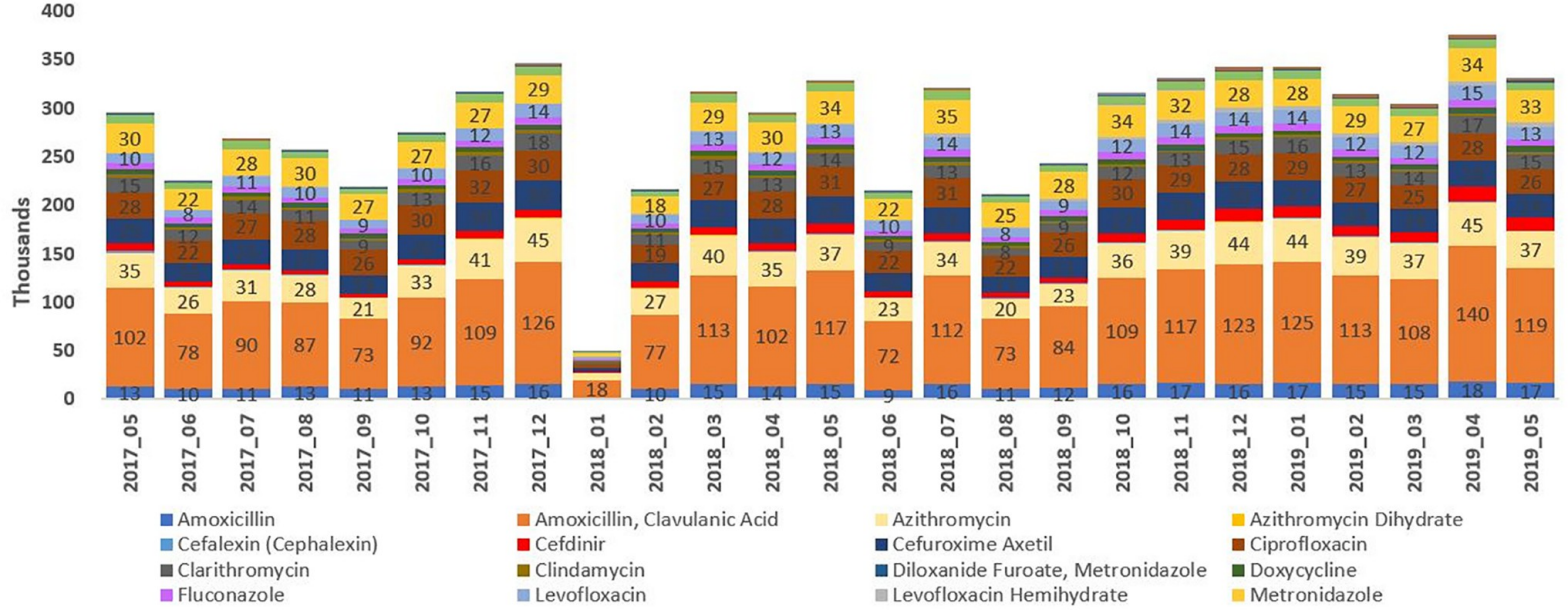
Antimicrobial claims (by molecule) in the year pre- and post-policy enforcement.

The new policy did not have any notable impact on the utilization pattern of different antimicrobials, by chemical classes and by molecules, in SA (Figs 3A, 3B, 4A, 4B and 5). Broad spectrum penicillin (J01C–ATC3) accounted for a large proportion of the sales volume and utilization in both the pre- and post-policy periods. Use of penicillin marginally decreased (by ~20–24%) in the post-policy period (sales: 11,648 thousand units; DDD: 71,039; DID: 2.88) versus the pre-policy period (sales: 14,700 thousand units; DDD: 91,227; DID: 3.78) (Table 1, Figs 3A and 4A). Further, the use of macrolides (J01F), fluoroquinolones (J01MA), and cephalosporin combinations (J01D) was high in both the periods (pre-policy period: sales: 5,579, 4,388, and 2,637 thousand units; DDD: 30,091, 23,392, and 23,002; DID: 0.43, 0.56, and 0.35 respectively; post-policy period: sales: 4,142, 4,032, and 2,389 thousand units; DDD: 24,688, 21,640, and 20,103; DID: 0.36, 0.56, and 0.29 respectively) (Table 1, Figs 3A and 4A). Sales, DDD and DID of amoxicillin-clavulanic acid combination, azithromycin, cefuroxime and amoxicillin alone were high in both the periods. The top three drugs by sales volume and utilization, were amoxicillin + clavulanic acid, amoxicillin, and metronidazole in the pre-policy period (sales: 10,781, 3,918, and 4,423 thousand units; DDD: 54,724, 36,502, and 36,502, DID: 4.54, 3.02, and 1.77, respectively) and post-policy period (sales: 9,306, 2,342, and 4,060 thousand units; DDD: 48,576, 22,462, and 19,972; DID: 3.93, 1.81, and 1.61, respectively) (Table 1, Figs 3B and 4B). The claims for amoxicillin-clavulanic acid combination, azithromycin, azithromycin dihydrate, ciprofloxacin, and cefuroxime were high during the pre-and post-policy periods (Fig 5).

## Discussion

This was the first study that objectively assessed the impact of the new legislative policy on the utilization of antimicrobials in the private retail sector (pharmacies) in SA. Overall, the antimicrobial sales and DDD (utilization) showed a slight decline during the post-policy period compared to the pre-policy period. Also, there was a drastic drop in sales, DDD and claims in the month preceding the month of enforcement, we speculate that this sudden drop in both parameters observed in April 2018 was in anticipation of the new policy, which was to be implemented in the next month (May 2018). We observed a sharp drop in sales and utilization in the immediate 2 to 4-month period after the policy enforcement in May 2018. Following this decline, both, the sales and DDD showed an overall rising trend, with values close to those recorded in the pre-policy period. The number antimicrobial claims increased after the policy activation, which suggests a decline in antimicrobial dispensation without prescriptions. The overall trends for sales, utilization and claims, between the pre- and the post-policy periods were however largely similar, with high utilization and claims for broad-spectrum penicillin, macrolides, cephalosporins and fluoroquinolones. The use of azithromycin, ciprofloxacin, cefuroxime and cefdinir was common, all of which belong to the WHO “Watch” group of antimicrobials [27]. To a large extent, this trend was consistent with the global antibiotic utilization data. According to a report published in 2014, broad spectrum penicillin and cephalosporins accounted for almost 55% of the total antimicrobial share [28]. This was also in line with the antimicrobial utilization data presented in the 2017 report for ESAC-Net countries, which showed that penicillin was the most frequently consumed antimicrobial in all countries across Europe, ranging from 33% to 67% of the total utilization in the community [29, 30]. The mean utilization for penicillin was 5.01 to 4.12 DID. Further, the mean utilization for cephalosporins (3.81 DID), macrolides (2.75 DID) and fluoroquinolones (2.16 DID) was also high in the European countries [30]. The mean antimicrobial utilization was relatively lower in the SA population (penicillin, 2.88–3.78 DID, cephalosporin, 0.29–0.35 DID, macrolides, 0.36–0.43 DID, fluoroquinolone, 0.56 DID).

In this study, a consistent pattern in the antimicrobial utilization was observed in the pre-policy and post-policy periods, with a peak in sales and DDD noted at the start of the autumn (beginning of October), which continued up to December, with another increase again in March. Similarly, an increase in the number of claims was observed between October and December in the pre-and post-policy periods. The indiscriminate over-the-counter use of antimicrobials for flu could have been a reason for such temporal association.

Even though antimicrobials were considered prescription-only medications before the new legislation, there were no provisions to punish the violators until then, which resulted in irrational prescribing practices by retail pharmacies. A survey in Makkah reported that 70% of the community pharmacists were not aware that dispensing antimicrobials without a prescription is illegal [31]. The current policy addresses this issue by imposing stricter punishments, in terms of hefty fines, imprisonment and revocation of the pharmacists’ licenses. The positive impact of such policies or laws, regulating antimicrobial dispensing has been reported in other studies, ensuring tighter control of antimicrobial dispensation and utilization [32–34]. For example, a study in Chile, showed significantly reduced sales and DDD of commonly prescribed antimicrobials after the introduction of a policy restricting dispensation of these drugs with only a legal prescription by an MD or DS [34].

Our study results indicate a positive impact of the new policy in curbing the utilization of antimicrobials in SA. However, this impact lasted for only 2–3 months following enforcement of the policy, and the sales, utilization and claims picked up thereafter, showing an increase in all these parameters in the post-policy period, albeit slightly lesser than the pre-policy values. The catching up of antimicrobial utilization from the decline at the immediate post-enforcement could be because patients had found a way to get prescription without proper examination or being diagnosed with bacterial infection by paying a nominal fee to the general practitioners. So, it is more of patient pressure and finding a back door through the system to get prescription for antimicrobials that possibly could have led to the increase in sales and utilization after the immediate decline post enforcement. The temporary and immediate decline following policy enforcement implies that controlling dispensing practices for antimicrobials in community pharmacies certainly had a positive impact. This new policy enforcing prescription-only access to antimicrobials would help reduce the rate of self-medication and compel patients to visit healthcare providers for consultation, thereby controlling malpractices such as hoarding antimicrobials for future use and encouraging family members or relatives to medicate themselves. These practices are prevalent in SA and other countries across the Middle East [1]. Further, the rise in antimicrobial utilization that was observed after 2–3 months of policy enforcement was unexpected and indicates that restricting non-prescription dispensing solely at the pharmacy level is not enough. Other key factors, such as behavioral aspects, perceptions and knowledge of patients and physicians also play a vital role in promoting the careful antimicrobial use, and therefore, it is essential to strike a balance across these complex interplaying factors [35, 36]. Many physicians in SA prescribe antimicrobials for viral infectious illnesses, either to avoid a secondary bacterial infection or due to a lack of awareness. Also, the over prescribing tendency of physicians is often related to the patients’/parents’ pressure [37]. Further, personal attitudes and beliefs of the general population potentially contribute to antimicrobial misuse. The misconception that antimicrobials are extremely effective in treating all kinds of infections, including viral infections, has largely increased self-medication, resulting in high rates of antibiotic resistance. Furthermore, patient-focused interventional strategies such as educational campaigns and awareness programs reiterating the detrimental effects of antimicrobial misuse and emphasizing the importance of finishing the entire course of antimicrobial treatment according to the physician’s recommendation are essential [38]. Also, interventional strategies focusing on improving doctor-patient rapport are important [36]. Moreover, educational/training programs for physicians, including those focusing on the antimicrobial usage guidelines would aid in reducing antimicrobial misuse. Published studies from Riyadh and Jeddah highlight the lack of awareness and reluctance to follow guidelines for antimicrobial use among physicians [36]. Furthermore, auditing prescribers (doctors) to assess their knowledge, perceptions, and antimicrobial prescribing patterns would be required to ensure correct diagnosis and appropriate selection of antimicrobials. Also, regular audits to track the implementation of policies and ensure long-term effectiveness are required.

Our study is the first in SA to include the use of DDD for calculating the utilization of antimicrobials in community settings. DDD is an universally accepted measurement unit endorsed by the WHO. It is used to express drug utilization and does not require patient-level data. DDD is an effective tool used to rank countries based on their drug utilization [39]. This is also the first study that quantifies the impact of this new policy on the utilization of antimicrobials in the private retail sector in SA.

There are a few limitations of this study that need to be acknowledged. Our analyses were based on antimicrobial sales to retailers (pharmacies) and did not include direct dispensation from the retail pharmacies. Therefore, these findings do not directly reflect the actual utilization of antimicrobials in the private sector. Nevertheless, we tried to compensate for this limitation through claims data analyses. Although DDD is an internationally recognized method, it does not allow back tracking of the utilization by an individual patient [40]. Regarding analyses of antimicrobial claims, data could be assessed for only one payer, which might limit the derived conclusions. Data were not interpretable for the other two payers: for one payer, we observed a decline in month-over-month claims, without any trend for antimicrobial utilization, whereas for the other, activity breakup (medication, procedures, consultation, services, consumables and others) for the year 2019 was not available. For further studies, prescription analysis and AMR monitoring can also be performed alongside antimicrobial utilization assessment, to facilitate informed clinical decision-making.

## Conclusion

The new policy has shown a temporary effect in decreasing the utilization of antimicrobials in the community pharmacy setting across SA. Further, legislations that mandate documentation of indication for the prescribed antimicrobials, measures such as audits, education, and training of physicians, and improving awareness of patients would help enhance the impact of such policies.

Overall, these findings will help to strengthen the objective efforts of the Saudi MoH in curbing antimicrobial misuse or overuse, by enforcing stringent regulatory control over the sales and dispensation of these life-saving drugs in the country.

## Supporting information

S1 Checklist STROBE. (DOCX)

S1 Data Data analysis. (XLSX)

## Abbreviations

ATC: Anatomical Therapeutic and Chemical
CLT: Central Limit Theorem
DDD: Defined Daily Dose
MDR: Multi-Drug Resistant
MoH: Ministry of Health
OTC: Over-The-Counter
RDD: Regression Discontinuity Design
SA: Saudi Arabia
WHO: World Health Organization
